# Artificial Intelligence and Health in Nepal

**DOI:** 10.3126/nje.v10i3.31649

**Published:** 2020-09-30

**Authors:** Alexander van Teijlingen, Tell Tuttle, Hamid Bouchachia, Brijesh Sathian, Edwin van Teijlingen

**Affiliations:** 1 Department of Pure and Applied Chemistry, Strathclyde University, Glasgow, UK; 2 Department of Computing & Informatics, Bournemouth University, Bournemouth, UK; 3 Geriatrics and long term care Department, Rumailah Hospital, Hamad Medical Corporation, Doha, Qatar; 4 Centre for Midwifery, Maternal and Perinatal Health, Bournemouth University, UK; 5 Centre for Disability Studies, Mahatma Gandhi University, Kerala, India; 6 Manmohan Memorial Institute of Health Sciences, Kathmandu, Nepal; 7 Nobel College, Kathmandu, Nepal

**Keywords:** Artificial Intelligence, Machine Learning, Big Data, Health, Nepal

## Abstract

The growth in information technology and computer capacity has opened up opportunities to deal with much and much larger data sets than even a decade ago. There has been a technological revolution of big data and Artificial Intelligence (AI). Perhaps many readers would immediately think about robotic surgery or self-driving cars, but there is much more to AI. This Short Communication starts with an overview of the key terms, including AI, machine learning, deep learning and Big Data.

This Short Communication highlights so developments of AI in health that could benefit a low-income country like Nepal and stresses the need for Nepal’s health and education systems to track such developments and apply them locally. Moreover, Nepal needs to start growing its own AI expertise to help develop national or South Asian solutions. This would require investing in local resources such as access to computer power/capacity as well as training young Nepali to work in AI.

## Introduction

The growth in information technology and computer capacity has opened up opportunities to deal with much and much larger data sets than even a decade ago. There has been a technological revolution of big data and Artificial Intelligence (AI). Perhaps many readers would immediately think about robotic surgery or self-driving cars, but there is much more to AI. This Short Communication starts with an overview of the key terms, including AI, machine learning, deep learning and Big Data.

AI as a discipline sets the goal of designing systems capable of reproducing the behaviour of humans in their reasoning activities. Such systems are usually referred to as intelligent systems. Often, such systems need a mixture of hardware and software to acquire and apply knowledge in an “intelligent” way and have the capabilities of perception, reasoning, learning, and making inferences from existing information. There are two types of AI, referred to as: strong and weak. Strong AI aims at developing systems that actually think and execute tasks in the way humans do. Weak AI aims at getting systems to work without figuring out how human reasoning works. AI consists of a number of branches, including: machine learning, natural language processing and understanding, pattern recognition, machine vision, robotics, computer audition, and speech recognition.

Machine learning (ML) is relevant and applicable to all branches for various purposes. As a subfield of AI, ML investigates the mechanisms by which knowledge is acquired through learning from experience. In other terms, ML focuses on designing algorithms/systems that learn from data. Such learning algorithms use sample data to generate a model which will be used for decision making in the future from subsequent data emanating from the same data source. We distinguish at least three paradigms of ML algorithms: supervised learning (where learning requires the presence of the desired output – called the ground truth), unsupervised learning (where training data does not have the desired output) and reinforcement learning (where learning follows from a sequence of actions informed by interaction with the environment).

Deep learning is a sub-class of ML methods that are largely based on neural networks and representation learning. Artificial neural networks are largely inspired by neural networks in the brain. Such networks are organised as layers of neurons interconnected by weighted links (synapses). So, the more layers there are, the deeper the network will be. Each layer transforms its input and produces an output that is sent to the next layer. The first layers in a deep network focus on the details at the smallest scales, while the following layers build coarse granularity by combining small-scale features at previous layers. The deeper the layers, the more they are able to represent abstract concepts. Think of learning to discriminate images. In a deep network architecture, the first layers might help isolate characteristic elements of object in the images, and the deeper layers will contribute to the emergence of the full object (e.g., person). Intermediate layers contribute to the formation of larger elements (nose, mouth, etc.) of the object. Deep learning is now considered as the most successful paradigm of ML and AI.

Finally, Big Data refers to huge amounts of data that are hard to examine in the traditional way. In its normative definition, Big Data is characterised by a set of five V’s: Volume (size), Velocity (rapid and continuous growth), Variety (structured and unstructured formats), Veracity (quality), and Value (usefulness). The concept of Big Data analytics is about learning, discovering and inferring knowledge from large (enormous) data sets.

AI algorithms have been successfully applied in various healthcare and medical domains as decision support tools for diagnosis to prognosis (predictive analysis) applications. For instance, medical imaging has been a target for AI tools, especially for deep learning as it is proven to be highly competitive to human medical performance. Application of deep learning has been investigated in a range of health fields and diseases, such radiomics, neurosurgical imaging, skin lesion, tumour, chest pain, neurologic diseases such as Alzheimer disease [1-2]. Other traditional AI and machine learning algorithms have also been applied to various diagnosis areas including breast cancer, drug discovery, therapy selection, and stratified care delivery. To enhance the efficiency of machine learning algorithms, substantially large datasets are required. This is particularly relevant to deep learning algorithms. The concept of (longitudinal) big data becomes quite appealing as data from different sources in various format (text, image, numerical, video) can be combined to make well-informed diagnosis or prognosis decisions.

These electronic health databases can allow for a personalized approach to medicine by improving diagnoses and predicting individual therapy responses, within clinical research and practice this would constitute a revolution if successful. Although the vision of personalized medicine is exciting, we would argue that it still has major challenges, many of which cannot be overcome by algorithmic ambiguity, and call for collaboration between traditional methodologists and medical ML experts to avoid considerable research waste [3].

### AI in Health Care

AI adds another dimension to the discipline of Epidemiology [4]. Epidemiology often uses large data sets to seek out the relative influence of a range of determinants on a patient group or population or to assess the prevalence and incidence of (rare) diseases.

AI and its sub-fields have impacted healthcare in many ways from diagnostics, triage, to treatment and planning of care. For example, the ability to predict a patient’s risk of having to undergo prolonged mechanical ventilation (PMV) can improve patient outcomes and reduce costs to intensive care units (ICUs). For example, Abujaber and colleagues [5] designed a ML method which is predictive of the duration a patient will need to undergo PMV after experiencing a traumatic brain injury (TBI), thus allowing doctors to design more appropriate individual care plans and make an earlier decision on whether to perform a tracheostomy which is a key factor in patient outcome.

This technological revolution could have a great impact on low-income countries like Nepal. Especially if AI produces low cost solutions that are otherwise too expensive for most people in a low-income country. We hope to present some examples of AI progress that can be employed in Nepal.

Birth asphyxia is one of the highest causes of new-born mortality worldwide with blood gas analysis of arterial blood being the confirmatory clinical diagnosis. However, this is an often-cost-prohibitive and unavailable technology in low-income countries resulting in a disproportionality high number of new-born asphyxia deaths occurring in low- and middle-income countries due the delayed diagnosis which comes after visual symptoms have emerged. Significant delay in diagnosing asphyxia and reversing may leave the patient with permanent brain damage. Ubenwa, a mobile phone app is being developed and tested in Nigeria which provides a machine learning algorithm (support vector classifier) which classifies new-born cries to identify asphyxia, this diagnostic tool provides the diagnosis with 20 seconds and allows an earlier intervention with only the requirement of a mobile phone, a tool rapidly growing in circulation within low income countries [6]. The model in Nigeria is trained on new-born cries recorded directly by doctors in Mexico [7] which goes to demonstrate the geographically transitive power of these tools. Diabetic eye disease is a very common diseases in Ophthalmology and is a growing public health issue. The incidence of vision-threatening diabetic retinopathy in high-income countries is declining, combining improved diabetic management and ophthalmological treatment, but globally it is increasing in low-income countries [8]. With findings that greatly enhance early detection and widening digital imaging (high resolution retinal photography and optical consistency tomography), combined with the potential for permanent morbidity, diabetic retinopathy is an excellent screening candidate. As far as we know it is the first medical device that is permitted to make a screening decision without clinician supervision, stratifying patients from those needing ophthalmology testing to patients who do not need yearly screening.

In dermatology recognizing visual pattern recognition is a key diagnostic skill, and AI has the potential to increase image perception and enhance diagnostic accuracy [9]. New neural computational networks have been used to diagnose skin disorders through visual image recognition, showing sometimes higher sensitivity and accuracy than professionally trained dermatologists. This field particularly can benefit from the work already carried in digital image processing and AI interpreted computer imagery.

In the current Covid-19 pandemic, many are seeking conventional as well as AI solutions [10]. One conventional Public Health measure is regular antibodies testing of a subset of a population. However, this is an expensive method confounded by the limit number of test kits the world can produce each day and how they are distributed across high, middle and low income countries. Online search trends are a possible pseudo-diagnosis of a population, of the Covid-19 symptoms and their associated probabilities of occurrence. Lampos et al. trained a transfer machine learning model (with Italian data to train each model prior to training on the relevant countries’ data). This model provides a good estimate of trend in number of daily confirmed cases in six countries [11]. This low budget tracking method provides a resource for low and middle-income nations to track the prevalence of the disease in their countries and take preventative measures, as well as evaluated past measures in accordance with the severity of the situation. Training an algorithm to predict outcomes such as death, admission to intensive care units or the need for mechanical ventilation can have a major clinical effect. ML algorithms and procedures are also versatile, new algorithms designed during this pandemic may be used for other respiratory diseases in the future.

In terms of patient care AI can be used to rapidly transfer knowledge between institutions on a global scale. An example of this is ultrasound, a widely used imaging method that is applied in a wide range of sub-specialties. High-quality ultrasound images are the basis of expert interpretation, however AI is helping to provide a route for capturing of images automatically, even uninitiated and has the potential to diagnose and monitor COVID-19 patients via ultrasound of the lungs, without having to teach health workers how to interpret results [12].

### Clinical Trials and AI

The inclusion of AI solutions in clinical trials is a relatively new phenomenon. The 2013 SPIRIT (Standard Protocol Items: Recommendations for Interventional Trials) statement aims to increase clinical trial reporting by offering evidence-based guidance on the minimum number of items to be addressed [13], in order to promote a transparent appraisal of new therapies. Like any new therapy, AI-based health interventions need to be systematically assessed to demonstrate their effectiveness. The SPIRIT-AI Extension is the recognized guideline for clinical trial protocols on AI-component interventions and includes 15 new essential elements. SPIRIT-AI encourages researchers to be transparent and clearly describe the AI intervention, including instructions and the required skills, the context in which the AI intervention will be implemented, data management issues, human-AI interaction and error case analysis. In addition, SPIRIT-AI will help journal editors, peer reviewers and readers to understand, evaluate and critically assess the design and risk of bias in a clinical trial.

## The way forward

In conclusion, there will be many AI solutions in health care currently under development or already in use elsewhere that can be applied to low-income countries. It is important for a country like Nepal to track such developments and apply them locally. This requires policymakers, health care providers (public and private) and universities to put an effort into staying up-to-date with global development in this field.

Secondly, we recommend that Nepal starts growing its own AI expertise to develop national or South Asian solutions. This would require local resources for example to access computer power/capacity as well as training young Nepali to work in AI. Studying epidemiology is good first step, but profiling and in-depth statistical analysis of large data set are different from machine learning.
